# A Meta-Analysis of Induced Abortion, Alcohol Consumption, and Smoking Triggering Breast Cancer Risk among Women from Developed and Least Developed Countries

**DOI:** 10.1155/2022/6700688

**Published:** 2022-11-16

**Authors:** Md. Akhtarul Islam, Nusrat Jahan Sathi, Hossain Mohammad Abdullah, Tarana Tabassum

**Affiliations:** Statistics Discipline, Science Engineering & Technology School, Khulna University, Khulna-9208, Bangladesh

## Abstract

**Background:**

The most prominent form of cancer in women is breast cancer, and modifiable lifestyle risk factors, including smoking, alcohol consumption, and induced abortion, can all contribute significantly to this disease.

**Objectives:**

This study's primary purpose was to assess the prevalence of breast cancer among women in developed and developing countries and the association between three modifiable hazard factors (induced abortion, smoking behavior, and alcohol use) and breast cancer.

**Methods:**

This study performed a systematic literature database review up to September 21, 2021. We employed meta-analytic tools such as the random effects model, forest plot, and subgroup analysis to conduct the research. Additionally, we conducted a sensitivity analysis to assess the influence of outliers.

**Results:**

According to the random effects model, smoker women have a higher risk of developing breast cancer from different countries (OR = 1.46; 95% CI: 1.08–1.97). In the case of induced abortion, the pooled estimate (OR = 1.25; 95% CI: 1.01–1.53) indicated a significant link between abortion and breast cancer. Subgroup analysis revealed that smoking substantially influences breast cancer in developing and developed countries. Breast cancer was more common among women who smoked in developed countries than in developing nations.

**Conclusion:**

The observed findings give sufficient support for the hypothesis that smoking and abortion have a significant influence on breast cancer in different nations. Health organizations should individually design comprehensive scientific plans to raise awareness about the risks of abortion and smoking in developed and developing countries.

## 1. Introduction

As the most commonly diagnosed neoplasm, breast cancer is a leading cause of cancer-related mortality among females in both developed and less developed nations [1, 2]. Cancer has spread to the majority of countries (154 out of 185) and is currently the primary cause of cancer-related deaths in more than 100 nations [3]. In conformity with the global cancer statistics for 2018, about 2.1 million recent cases, representing nearly one of every four women, were diagnosed with breast cancer. Approximately 626,679 women died due to breast cancer in 2018 [4]. The incidence (number of new cases occurring or rate per 100,000 persons per year) is highest in developing countries, which account for 60% of the deaths, yet it is growing at a faster pace in middle- and low-income countries [5, 6]. More specifically, most occurrence rates are detected in many European countries, notably Switzerland, Italy, and U.S. whites, whereas rates are low in South America, Asia, and Africa [7]. The incidence rate for women living in developed countries (except Japan) is four times higher than that of the least developed countries [8, 9]. A risk factor is defined as an element that increases the probability of inciting breast cancer [10]. In this way, the identification of modifiable breast cancer risk factors has crucial implications for preventing and reducing the incidence of breast cancer [11]. Physical activity, diet, weight, use of oral contraceptives, alcohol, ingestion of smoke, anxiety, and stress are conventionally modifiable risk factors [12]. Alcohol consumption and smoking are modifiable influencing factors that are generally related to breast cancer to a few more extensive degrees [13, 14]. Besides, it is grounded that full-term pregnancy (without abortion or miscarriage) consummately recommends a defensive impact on the possibility of breast cancer. In contrast, the idea of incomplete pregnancies affecting the risk of breast cancer remains ambiguous [15]. Various articles have explored the association between alcohol consumption, smoking intake, induced abortion, and breast cancer [16–31]. Previous research suggested an association between alcohol consumption and breast cancer [16–18, 21–24]. Moreover, it is evident that multiple studies have found a possible link between smoking and breast cancer [25, 29, 32, 33]. Alcohol causes approximately 4% of breast cancer cases in developed nations [32]. Numerous research studies have suggested a beneficial relationship between breast cancer and induced abortion. Regardless of the alarmingly high frequency of breast malignancy and prompted abortion, the past forty years have delivered neither agreement of opinion into the clinical research nor a need to keep moving to show up at one. Nevertheless, several studies have shown an inverse, null, or weak association between breast cancer and these risk factors (alcohol consumption, smoking intake, and induced abortion), leading to inconsistent findings [15, 18–20, 26, 34–40]. It may be owing to the short sample size and methodological constraints [36]. Moreover, biases, especially those connected with the case-control studies and the insufficient alternative of the reference group, can produce conflicting results on induced abortion and breast cancer [41]. The literature review reveals that the association between three lifestyle-related variables (such as abortion, alcohol consumption, and smoking) and breast cancer varies between studies. The generalization of lifestyle-related indicators' influence on breast cancer among women is pivotal in light of their clinical significance, although it is scarce in the literature. To overcome this gap, the primary aim of this study is to apply a meta-analysis based on a comprehensive review of observational studies published by 2021. This study elucidates the degree of association between these three attributes and breast cancer among women from least developed and developed countries.

## 2. Methods

### 2.1. Data Source and Search Strategy

The previous works of literature were individually searched in four English databases (PubMed, Wiley, Scopus, and ScienceDirect) and most commonly searched in Google Scholar. These searches are conducted manually. The searching strategy utilized different search keywords in each database: (1) “breast cancer,” “breast carcinoma,” “breast tumor,” “breast neoplasm,” “mammary cancer,” “mammary carcinoma,” “mammary neoplasm,” “smoking,” “alcohol consumption,” and “abortion.” (2) “Breast cancer,” “smoking,” “alcohol consumption,” and “abortion.” (3) “Risk,” “risk factors,” “influencing factors,” “susceptibility,” phrased with “breast cancer,” “smoking,” “alcohol consumption,” and “abortion.” (see supplementary file 1). Therefore, the search strategy required four stages for this potential study: (1) cross-sectional study, cohort study, prospective study, and case-control study. (2) Breast cancer, mammary carcinoma, breast neoplasm, breast carcinoma, mammary neoplasm, mammary cancer, and breast tumor. (3) Risk, risk factors, influencing factors, and susceptibility. (4) Name of the particular country.

We considered literature in the present investigation based on the following criteria: (a) bivariate data available for the breast cancer risk with alcohol consumption, smoking influence, and abortion cases; (b) article full-text availability; (c) information made available in the English language; and (d) peer-reviewed, accepted, or published articles. The authors evaluated the appropriateness of the studies after finding the full texts. In the case of multiple studies in one country, the data of individual variables were appropriately merged.

### 2.2. Study Selection and Data Extraction

The following criteria for including different studies were identified in accordance with the PICOs acronym:

  Population: women with breast cancer.
  Intervention: consider three lifestyle-related indicators (e.g., abortion, alcohol consumption, and smoking) of developing breast cancer.
  Comparison: consider three lifestyle-related indicators (e.g., abortion, alcohol consumption, and smoking) of developing breast cancer.
  Outcomes: breast cancer, mammary neoplasm, breast neoplasm, breast tumor, mammary cancer, breast carcinoma, and mammary carcinoma.
  Study design: prospective study, cross-sectional study, cohort study, and case-control study.

**Figure 1:**
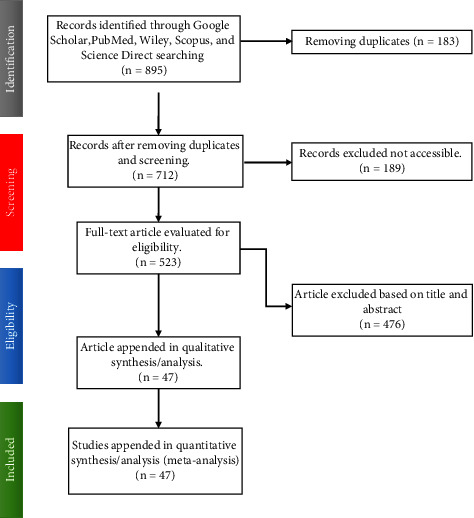
Flowchart illustrating the method for determining and including articles in the random effects meta-analysis.

Initially, 895 articles were appended after employing distinct search strategies and PICOs schema for each database. In the final stage, the authors rechecked and rescanned the abstracts of the included papers to ensure their accuracy.  Figure 1 depicts the overall eligibility requirements of the studies for the final assessment.

### 2.3. Statistical Analysis

We have applied the software R version 3.6.2 (Bell Laboratories, New Jersey, USA) and IBM SPSS version 27 (SPSS Inc., Chicago, USA) to convey the investigation. We utilized meta-analysis to examine studies from different countries. Computing values evaluated heterogeneity using the *p* values and *I*^2^ of the datasets [42, 43]. We performed the meta-analytical procedure by executing a random-effects model as this study found significant heterogeneity, which assessed DerSimonian and Laird's pooled effect [44]. The Q statistic, a weighted squared deviation, is used to estimate *I*^2^, and the value ranges from 0 to 100% [45] to display the 95% confidence interval, summary measure, and weight for each article for the most significant factors [46]. A leave-one-out sensitivity analysis was conducted to determine the effect of heterogeneity and outliers [47]. We utilized the odds ratio for the summary measures, and all outcomes were weighted to handle bias due to underselection and overselection [48]. For the dichotomous variable, the odds ratio (OR) as well as effect size were estimated with 95 percent confidence intervals (CI). A contour-enhanced funnel plot is adopted for the assessment of publication bias. We have observed the symmetry of the plot to determine whether there is a presence or absence of publication bias. In addition, Egger's test was used to estimate the risk of publication bias, with *p* values of 0.05 indicating the occurrence of publication bias [49].

### 2.4. Variables

In this meta-analysis, we well-thought-out breast cancer as the dependent variable. In addition, Egger's test was used to estimate the risk of publication bias, with *p* values of 0.05 indicating the occurrence of publication bias. We also considered the impacting factors of alcohol consumption, smoking, and abortion cases included as covariates to execute the exploration and find out the most impacting factors around the world.

## 3. Results

Table 1 represents the baseline characteristics of different selected studies focusing on smoking, alcohol consumption, and induced abortion triggering breast cancer among women of different countries.

Table 2 shows the output of the heterogeneity test for alcohol consumption. The estimated value of tau square is 0.25, which indicates the absolute estimated value of the between-study variation. From the value of *I*^2^, we have come to know that 95.2% of the overall variation is due to true heterogeneity (which can be explained). Also, the observed weighted value of S.S. is 456.00 with df = 22 and *p* value <0.001, thus significant.

Table 2 shows that the pooled estimate is 0.9401 and the 95% confidence interval is [0.751; 1.176]. This outcome suggests that alcohol consumption has no significant impact on breast cancer in different studies in different countries.

Table 3 shows the output of the heterogeneity test for smoking influence. The estimated value of the tau square is 0.55, which indicates the absolute estimated value of the between-study variation. From the value of *I*^2^, we have come to know that 98.8% of the overall variation is due to true heterogeneity (which can be explained). Also, the observed weighted value of S.S. is 1930.79 with df = 24 and *p* value <0.001, which is significant.

Table 3 shows that the pooled estimate is 1.454 and the 95% confidence interval is [1.08, 1.97]. This table suggests that smoking has an impact on breast cancer in different studies. The studies by Croghan et al., 2009; Ellingjord-Dale et al., 2017; Kaufman et al., 2008; and Liu et al., 2017, show the odds of breast cancer occurring due to smoking are the highest.

**Figure 2:**
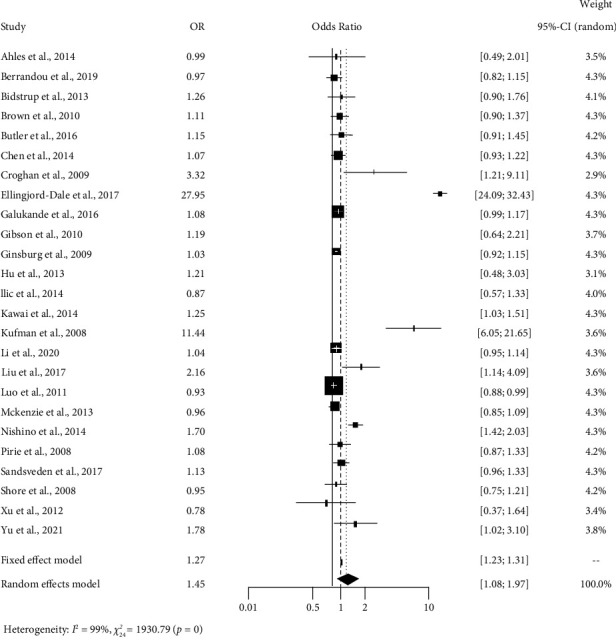
Forest plot showing the smoking influence on breast cancer.

Figure 2 shows the vibrant sight of the random effects model for variable smoking. Inclusive concise information on data from individual studies is given there. We can perceive individual studies' confidence intervals and estimated values with a rectangular shape and combined effects with a diamond shape. The combined effect for the fixed effects model is 1.27, and for the random effect, the model is 1.46. The overall visualization of the studies suggests that smoking significantly impacts breast cancer in different studies.

Table 4 shows the output of the heterogeneity test for abortion cases. The estimated value of tau square is 0.18. It indicates the absolute estimated value of the between-study variation. From the value of *I*^2^, we have come to know that 84.7% of the overall variation is due to true heterogeneity (which can be explained). Also, the observed weighted value of S.S. is 117.96 with df = 18 and *p* value <0.001, which is also significant.

Table 4 shows that the pooled estimate is 1.25, and the 95% confidence interval is [1.01; 1.53]. This table suggests that abortion case has an impact on breast cancer in different studies. The studies by Ahmed et al., 2015, and Balekouzou et al., 2017, have the highest odds of breast cancer occurring due to abortion cases.

**Figure 3:**
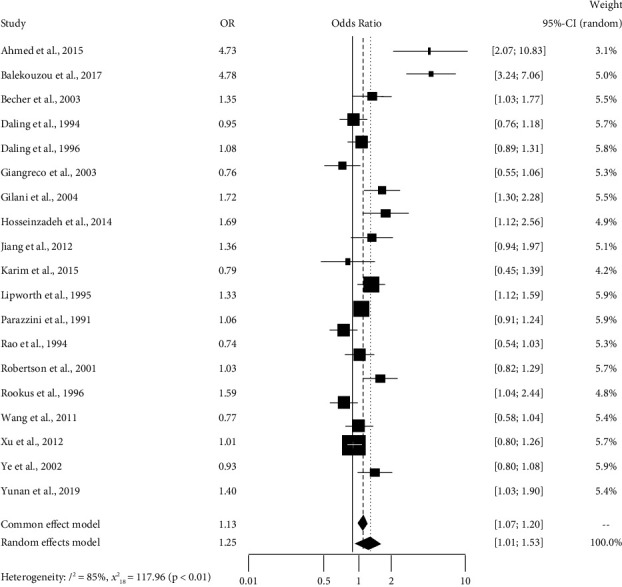
Forest plot showing the induced abortion case on breast cancer.

Figure 3 shows the vibrant sight of the random effects model for the variable abortion case. A comprehensive summary of the data from individual studies is given there. We can perceive individual studies' confidence intervals and estimated values with rectangular and combined diamond-shaped effects. The combined effect for the fixed effects model is 1.13, and for the random effects model, it is 1.25. The overall visualization of studies suggests that abortion cases significantly impact breast cancer in different studies.

**Figure 4:**
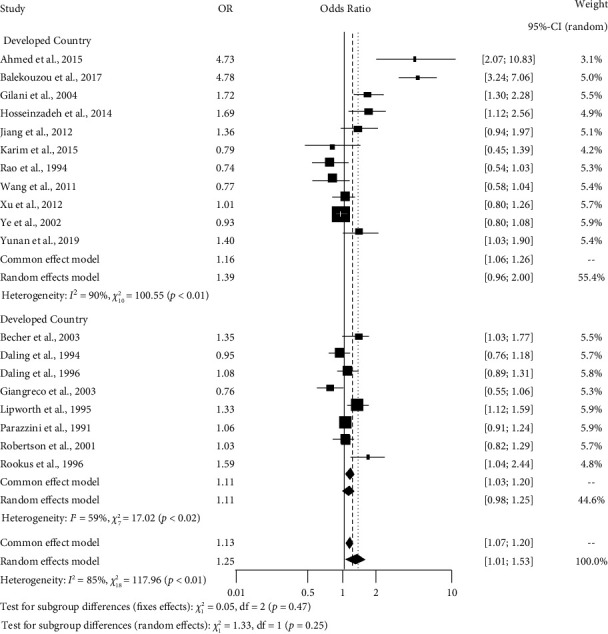
Forest plot showing subgroup analysis expressing the influence of induced abortion case by country status (developing or developed) on breast cancer.

**Figure 5:**
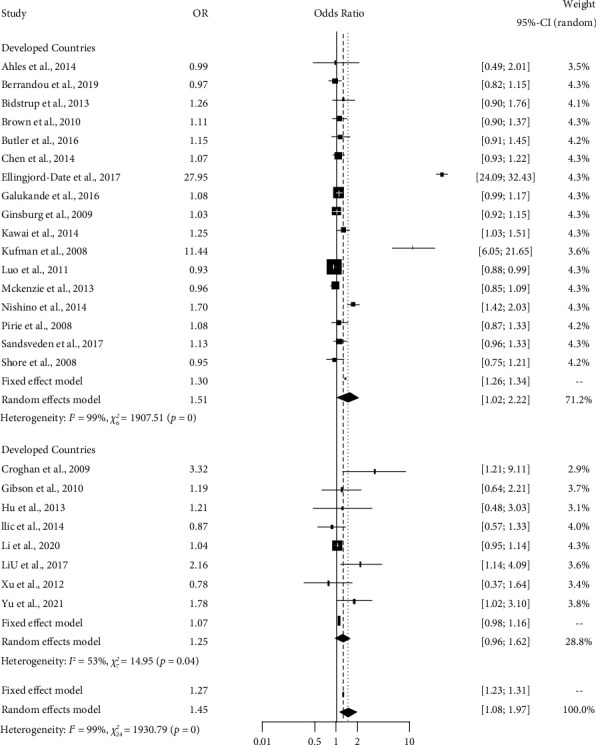
Forest plot showing subgroup analysis expressing the influence of smoking by country status (developing or developed) on breast cancer.

Table 5 represents that the cases of abortion and smoking have a substantial influence on breast cancer in developing and developed countries. Women who had abortions in developing countries were more likely to have breast cancer (OR: 1.39, *p* < 0.01, *I*^2^ = 90%) compared to women in developed countries (Figure 4). Besides, the odds of having breast cancer were higher among smoker women residing in developed countries (OR: 3.66, *p* < 0.01, *I*^2^ = 87%) than in women who smoked in developing countries (Figure 5).

**Figure 6:**
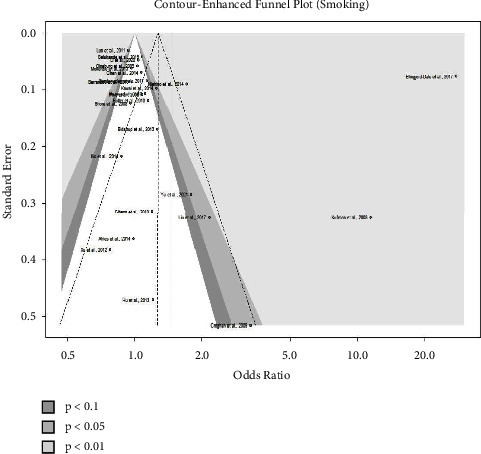
Contour-enhanced funnel plot of all studies of smoking.

**Figure 7:**
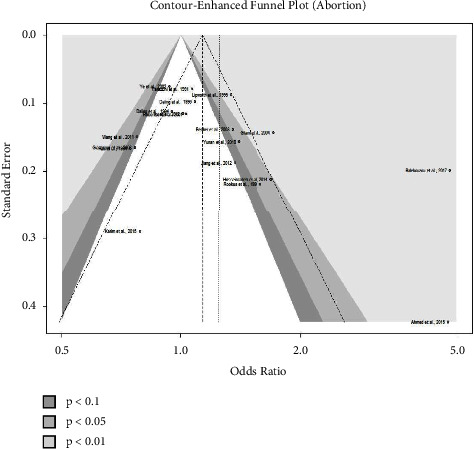
Contour-enhanced funnel plot of all studies of abortion.

At the 5% level of significance, Egger's test for a regression intercept produced nonsignificant *p* values of 0.3694 (smoking) and 0.0884 (abortion). It implies that there is no asymmetry in the funnel plot, which is compatible with the absence of publication bias. Therefore, the funnel plots depicted in Figures 6 and 7 show no evidence of publication bias.

## 4. Discussion

The purpose of this study is to systematically identify the degree of association between three lifestyle-related indicators (e.g., abortion, smoking, and alcohol consumption) and breast cancer risk in women in developed and least developed countries. Based on a systematic review of observational studies published in 2020 in PubMed, Wiley, and ScienceDirect, the study was analyzed. According to the author's best knowledge, this is one of the first studies to execute a meta-analysis of tracking breast cancer risk using three lifestyle-related indicators. The random effects model in the meta-analysis found that exposure to smoking and abortion was significantly related to the chance of developing breast cancer.

Women who smoked had a 45 percent greater likelihood of having breast cancer than women who did not smoke. Smoking appears to raise the chance of developing breast cancer in both developed and developing countries. The positive relationship between smoking and breast cancer that was discovered in the present studies was consistent with previous research [50–53]. The increased risk of breast cancer associated with smoking could be responsible for the impaired metabolic and immune systems compared to nonsmokers. For instance, a previous study mentioned that tobacco smoke had a substantial adverse influence on endocrine function [50]. This might have also contributed to having worse steroid-responsive tissues and a decreased rate of endometrial neoplasia, accounting for smoking as a human carcinogen.

Individuals with a history of abortion were also found to have an increased chance of developing breast cancer. A meta-analysis reached a similar conclusion, indicating that abortion increases women's risk of breast cancer [54]. Earlier studies that support this assertion have also found a statistically significant relationship between abortion and breast cancer risk [55, 56]. Contrary to this finding, two recent studies showed that women who do abortions have no influence on developing breast cancer [57–59]. The conflict could be due to variations in the environment, information, methodology, and so on. The precise data for abortion is arduous to gather as it is a very private incident for every individual. Therefore, it is argued that the combined effects of several articles increased the validity and accuracy of the present study findings.

In keeping with the findings of past systematic reviews, this investigation found no statistically significant relationship between alcohol use and breast cancer risk [60, 61]. The underreporting or absence of alcohol consumption in religious countries is one of the key factors explaining the absence of a relationship between alcohol consumption and breast cancer risk. In the literature, however, there were inconsistent findings about the relationship between alcohol use and breast cancer risk [62, 63]. Arguably, the inconsistency may be explained by the prevalence, dose, and type of alcohol consumption due to its non-normative patterns [61, 64]. Thus, because the present study used the most recently published articles, the influence of diverse alcohol consumption incidents varied from country to country. However, some biological factors are correspondingly impactful in this regard. Therefore, further research is required on a large scale to identify the effects of different types of alcohol consumption and treatments on breast cancer risk.

This current study also includes a subgroup analysis to demonstrate the effects of abortion and smoking on breast cancer in developing and developed countries. The risk of breast cancer is greater across developing territories because of abortion than in developed countries, consistent with an earlier study [65]. The nonutilization and unavailability of contraceptives among women in developing countries are observed, which increases the abortion rate [66]. Therefore, this discrepancy occurs due to birth control awareness restrictions in developing and developed settings. Besides, smoking is a sensitive factor in breast cancer in developed countries compared to developing countries. A study conducted with data from 187 countries similarly reveals that smoking influences breast cancer [67]. The possible reason might be that antismoking laws like MPOWER measures are not strictly followed in developed countries, provoking the increased possibility of smoking [68].

Smoking and abortion are two risk factors for breast cancer among women in developed and least developed countries, as shown in the present study. Strengthening the implementation of MPOWER policies might help create awareness among women about the hazards of smoking. In addition, multifaceted interventions like government, nongovernment, and NGO's health programs based on sexuality education, unintended pregnancy awareness, and effective contraception and emergency contraception are needed to reduce abortion in society, thus controlling the risk of breast cancer. Besides, comprehensive science-based strategies for developed and developing countries might be designed individually to create awareness about the risks of abortion and smoking.

Thus, smoking and induced abortion are connected with breast cancer in different nations, which has clinical significance. Its explication will aid health organizations and stakeholders in establishing comprehensive scientific plans to promote awareness about the risks of abortion and smoking in women. This agreement is supported by the extant literature [69, 70]. A study on breast cancer patients determined that awareness of the benefits of quitting smoking is related to a reduction in breast cancer severity [69].

## 5. Strength and Limitation

There are numerous unique strengths in the present study. Firstly, the methodology is the main advantage, as the systematic reviews combine findings from several published studies and draw a pooled conclusion from them. Secondly, this study considered three exposures to identify their relationship with breast cancer risk. Thirdly, subgroup analysis appends an additional advanced dimension to the current study.

The current study is not without limitations. Firstly, the methodology follows observational trials that restrict the nature of the generalizability of the study findings [45]. Secondly, the unavailability of factors such as genetic factors or family factors was not appended, which might contribute to the risk of breast cancer. Additionally, underreporting or the absence of alcohol consumption in religious countries could introduce bias into the study.

## 6. Conclusion

Initially, the risk of breast cancer was not associated with smoking-related cancer. Over time, however, sufficient evidence has accumulated to suggest that smoking is correlated with an increased risk of breast cancer. Although this study found no correlation between drinking and breast cancer, it did find a substantial association between induced abortion and breast cancer. This study reveals that the risk of breast cancer linked to smoking is higher in developed nations than in developing countries. So, the authority should consider these influences and make their strategies to raise awareness accordingly among people to reduce the smoking habit for a better healthcare situation in their respective countries.

## Figures and Tables

**Table 1:** Baseline characteristics of different selected studies for alcohol consumption, induced abortion, and smoking.

Study	Country	Country type	Breast cancer among women in the alcohol, abortion, or smoking group	Total number of women with alcohol, abortion, or smoking	Breast cancer among women with no alcohol, abortion, or smoking	Total number of women with no alcohol, abortion, or smoking
*Alcohol consumption*						
Ahles et al., 2014	USA	Developed country	136	288	18	28
Ahmed et al., 2015	Bangladesh	Developing country	28	60	52	100
Berrandou et al., 2019	France	Developed country	924	1899	201	398
Bidstrup et al., 2013	Denmark	Developed country	429	22660	19	1168
Brown et al., 2010	USA	Developed country	357	942	234	609
Butler et al., 2016	USA	Developed country	1242	2299	565	1071
Chen et al., 2014	China	Developing country	32	52	637	1299
Croghan et al., 2009	USA	Developed country	576	1225	3165	4072
Ellingjord-Dale et al., 2017	Norway	Developed country	3677	23586	725	5306
Galukande et al., 2016	Uganda	Developing country	31	135	77	208
Gibson et al., 2010	Philippines	Developing country	8	91	115	993
Hu et al., 2013	China	Developing country	18	34	178	373
Kawai et al., 2014	USA	Developed country	733	1439	220	447
Kufman et al., 2008	USA	Developed country	61	196	11	79
Li et al., 2020	21 centers in western countries	Developing countries	2175	6197	631	1786
Liu et al., 2017	China	Developing country	15	27	1466	2937
Nishino et al., 2014	Japan	Developed country	322	1093	815	3103
Pakzad et al., 2020	Iran	Developing countries	892	1863	40	69
Pirie et al., 2008	UK	Developed country	1082	1316	667	814
Sandsveden et al., 2017	Sweden	Developed country	1122	2193	64	161
Tong et al., 2014	China	Developing country	64	177	248	447
Xu et al., 2012	China	Developing country	37	135	379	1437
Yu et al., 2021	China	Developing country	123	224	910	1845

*Induced abortion*						
Ahmed et al., 2015	Bangladesh	Developing country	30	39	50	121
Balekouzou et al., 2017	Central African Republic	Developing country	114	213	60	309
Becher et al., 2003	Germany	Developed country	103	259	482	1465
Daling et al., 1994	USA	Developed country	193	422	652	1384
Daling et al., 1996	USA	Developed country	314	585	779	1506
Giangreco et al., 2003	USA	Developed country	74	168	670	1320
Gilani et al., 2004	Pakistan	Developing country	105	327	194	899
Hosseinzadeh et al., 2014	Iran	Developing country	64	157	76	263
Jiang et al., 2012	China	Developing country	76	140	284	610
Karim et al., 2015	Saudi Arabia	Developing country	47	104	45	88
Lipworth et al., 1995	Greece	Developed country	502	1341	318	1027
Parazzini et al., 1991	Italy	Developed country	423	789	1682	3230
Rao et al., 1994	India	Developing country	71	168	593	1195
Robertson et al., 2001	Slovenia	Developed country	247	490	377	758
Rookus et al., 1996	Netherlands	Developed country	56	92	862	1744
Wang et al., 2011	China	Developing country	125	273	275	527
Xu et al., 2012	China	Developing country	233	878	183	694
Ye et al., 2002	China	Developing country	344	135462	358	131574
Yunan et al., 2019	China	Developing country	355	694	93	217

*Smoking*						
Ahles et al., 2014	USA	Developed countries	74	100	49	66
Berrandou et al., 2019	France	Developed countries	437	901	688	1396
Bissonauth et al., 2009	Canada	Developed countries	178	358	102	232
Bidstrup et al., 2013	Denmark	Developed countries	124	5883	334	17544
Brown et al., 2010	USA	Developed countries	162	400	429	1151
Butler et al., 2016	Carolina, USA	Developed countries	866	1590	942	1782
Chen et al., 2014	China	Developing countries	16	21	653	1330
Croghan et al., 2009	USA	Developed countries	1278	1545	958	6552
Ellingjord-Dale et al., 2017	Norway	Developed countries	1098	6924	1748	11748
Gibson et al., 2010	Philippines	Developing countries	13	100	110	988
Ginsburg et al., 2009	USA	Developed countries	995	1975	1543	3101
Hu et al., 2013	China	Developing countries	10	19	186	388
Ilic et al., 2014	Serbia	Developing countries	61	128	130	254
Kawai et al., 2014	USA	Developed countries	354	653	606	1245
Kufman et al., 2008	USA	Developed countries	79	173	13	190
Li et al., 2020	21 centers in western countries	Developing countries	1330	3738	1476	4245
Liu et al., 2017	China	Developing countries	30	44	1455	2922
Luo et al., 2011	USA	Developed countries	1692	41022	3520	79990
Mckenzie et al., 2013	New Zealand	Developed countries	942	2294	856	2037
Nishino et al., 2014	Japan	Developed countries	242	650	918	3547
Prescott et al., 2007	USA	Developed countries	737	917	991	1252
Sandsveden et al., 2017	Sweden	Developed countries	680	1325	488	1011
Shore et al., 2008	USA	Developed countries	265	543	253	506
Xu et al., 2012	China	Developing countries	9	41	407	1531
Yu et al., 2021	China	Developing countries	35	55	1000	2016

**Table 2:** Summary of the random effects and fixed effects models for alcohol consumption.

Author	Country	OR	95% CI of OR	Fixed effects model (%)	Random effects model (%)
Ahles et al., 2014	USA	0.50	[0.22; 1.11]	0.4	3.1
Ahmed et al., 2015	Bangladesh	0.81	[0.43; 1.53]	0.5	3.6
Berrandou et al., 2019	France	0.93	[0.75; 1.15]	4	4.9
Bidstrup et al., 2013	Denmark	1.17	[0.73; 1.86]	0.8	4.2
Brown et al., 2010	USA	0.98	[0.79; 1.21]	4.2	4.9
Butler et al., 2016	USA	1.05	[0.91; 1.22]	8.4	5.1
Chen et al., 2014	China	1.66	[0.94; 2.94]	0.4	3.9
Croghan et al., 2009	USA	0.25	[0.22; 0.29]	18.3	5.1
Ellingjord-Dale et al., 2017	Norway	1.17	[1.07; 1.27]	23.6	5.1
Galukande et al., 2016	Uganda	0.51	[0.31; 0.83]	1.1	4.1
Gibson et al., 2010	Philippines	0.74	[0.35; 1.56]	0.4	3.3
Hu et al., 2013	China	1.23	[0.61; 2.49]	0.3	3.4
Kawai et al., 2014	USA	1.07	[0.87; 1.33]	3.9	4.9
Kufman et al., 2008	USA	2.79	[1.38; 5.65]	0.3	3.4
Li et al., 2020	21 centers in western countries	0.99	[0.89; 1.11]	15	5.1
Liu et al., 2017	China	1.25	[0.59; 2.69]	0.3	3.2
Nishino et al., 2014	Japan	1.17	[1.01; 1.37]	7.1	5
Pakzad et al., 2020	Iran	0.67	[0.41; 1.08]	0.9	4.2
Pirie et al., 2008	UK	1.02	[0.81; 1.28]	3.5	4.9
Sandsveden et al., 2017	Sweden	1.59	[1.15; 2.20]	1.4	4.7
Tong et al., 2014	China	0.45	[0.32; 0.65]	2.1	4.6
Xu et al., 2012	China	1.05	[0.71; 1.57]	1.1	4.4
Yu et al., 2021	China	1.25	[0.95; 1.65]	2.1	4.8
Pooled (random)		0.94	[0.75; 1.18]	100%	100%
$\hat{Q}$	456.00				
*df*	22				
*P* − *value*	<0.0001				
${\hat{I}}^{2}$	95.2%				
${\hat{\tau }}^{2}$	0.25				

OR odds ratio; CI confidence interval.

**Table 3:** Summary of the random effects and fixed effects models for smoking.

Author	Country	OR	95% CI	Fixed effects model (%)	Random effects model (%)
Ahles et al., 2014	USA	0.99	[0.49; 2.01]	0.2	3.5
Berrandou et al., 2019	France	0.97	[0.82; 1.15]	3.7	4.3
Bissonauth et al., 2009	Canada	1.26	[0.90; 1.76]	0.8	4.1
Bidstrup et al., 2013	Denmark	1.11	[0.90; 1.37]	2.2	4.3
Brown et al., 2010	USA	1.15	[0.91; 1.45]	1.7	4.2
Butler et al., 2016	Carolina, USA	1.07	[0.93; 1.22]	5.3	4.3
Chen et al., 2014	China	3.32	[1.21; 9.11]	0.1	2.9
Croghan et al., 2009	USA	27.95	[24.09; 32.43]	0.8	4.3
Ellingjord-Dale et al., 2017	Norway	1.08	[0.99; 1.17]	14.4	4.3
Gibson et al., 2010	Philippines	1.19	[0.64; 2.21]	0.2	3.7
Ginsburg et al., 2009	USA	1.03	[0.92; 1.15]	7.9	4.3
Hu et al., 2013	China	1.21	[0.48; 3.04]	0.1	3.1
Ilic et al., 2014	Serbia	0.87	[0.57; 1.33]	0.6	4
Kawai et al., 2014	USA	1.25	[1.03; 1.51]	2.5	4.3
Kufman et al., 2008	USA	11.44	[6.05; 21.65]	0.1	3.6
Li et al., 2020	21 centers at western countries	1.04	[0.95; 1.14]	11.7	4.3
Liu et al., 2017	China	2.16	[1.14; 4.09]	0.2	3.6
Luo et al., 2011	USA	0.94	[0.88; 0.99]	30.2	4.3
Mckenzie et al., 2013	New Zealand	0.96	[0.85; 1.09]	7	4.3
Nishino et al., 2014	Japan	1.70	[1.43; 2.03]	2.4	4.3
Prescott et al., 2007	USA	1.08	[0.87; 1.33]	2.2	4.2
Sandsveden et al., 2017	Sweden	1.13	[0.96; 1.33]	3.6	4.3
Shore et al., 2008	USA	0.95	[0.75; 1.22]	1.8	4.2
Xu et al., 2012	China	0.78	[0.37; 1.64]	0.2	3.4
Yu et al., 2021	China	1.78	[1.019; 3.10]	0.3	3.8
Pooled (random)		1.46	[1.08; 1.97]	100%	100%
$\hat{Q}$	1930.79				
*df*	24				
*P* − *value*	0.000				
${\hat{I}}^{2}$	98.8%				
${\hat{\tau }}^{2}$	0.55				

OR odds ratio; CI confidence interval.

**Table 4:** Summary of the random effects and fixed effects models for induced abortion.

Author	Country	OR	95% CI	Fixed effect (%)	Random effect (%)
Ahmed et al., 2015	Bangladesh	4.73	[2.07; 10.83]	0.3	3.1
Balekouzou et al., 2017	Central African Republic	4.78	[3.24; 7.06]	1.0	5.0
Becher et al., 2003	Germany	1.35	[1.03; 1.77]	3.9	5.5
Daling et al., 1994	USA	0.95	[0.76; 1.18]	7.4	5.7
Daling et al., 1996	USA	1.08	[0.89; 1.31]	9.1	5.8
Giangreco et al., 2003	USA	0.76	[0.55; 1.06]	3.8	5.3
Gilani et al., 2004	Pakistan	1.72	[1.30; 2.28]	3.2	5.5
Hosseinzadeh et al., 2014	Iran	1.69	[1.12; 2.56]	1.5	4.9
Jiang et al., 2012	China	1.36	[0.94; 1.97]	2.2	5.1
Karim et al., 2015	Saudi Arabia	0.79	[0.45; 1.39]	1.2	4.2
Lipworth et al., 1995	Greece	1.33	[1.12; 1.59]	10.1	5.9
Parazzini et al., 1991	Italy	1.06	[0.91; 1.24]	13.8	5.9
Rao et al., 1994	India	0.74	[0.54; 1.03]	3.8	5.3
Robertson et al., 2001	Slovenia	1.03	[0.82; 1.29]	6.6	5.7
Rookus et al., 1996	Netherlands	1.59	[1.04; 2.44]	1.5	4.8
Wang et al., 2011	China	0.77	[0.58; 1.04]	4.6	5.4
Xu et al., 2012	China	1.01	[0.80; 1.26]	6.7	5.7
Ye et al., 2002	China	0.93	[0.80; 1.08]	16.3	5.9
Yunan et al., 2019	China	1.40	[1.03; 1.90]	3.1	5.4
Pooled (random)		1.25	[1.01; 1.53]	100%	100%
$\hat{Q}$	117.96				
*df*	18				
*P* − *value*	<0.0001				
${\hat{I}}^{2}$	84.7%				
${\hat{\tau }}^{2}$	0.18				

**Table 5:** Summary of the subgroup for abortion case and smoking random effects analysis and fixed effects model for abortion.

Variables	Developing countries		Developed countries	
	Or (95% CI)	*p* value **I**^2^	Or (95% CI)	*p* value **I**^2^
Abortion cases	1.39 [0.96; 2.00]	<0.01 90%	1.11 [0.98; 1.25]	<0.01 59%
Smoking	2.92 [2.25; 3.79]	<0.01 86%	3.66 [2.95; 4.55]	<0.01 87%

*Note. Q*. heterogenic statistic; *I*^2^ between study variation; OR. odds ratio; CI. confidence interval.

## Data Availability

The data supporting the findings of this study are available from the corresponding author upon request.

## Supplementary Materials

Supplementary Materials S1: a comprehensive search strategy for each database.
