# Supplementary material for: A Meta-Analysis of Induced Abortion, Alcohol Consumption, and Smoking Triggering Breast Cancer Risk among Women from Developed and Least Developed Countries
Source: Int J Clin Pract. 2022 Nov 16;2022:6700688. doi: 10.1155/2022/6700688 (PMC9683974; doi:10.1155/2022/6700688)
Supplement: Supplementary Materials — S1: a comprehensive search strategy for each database. [file 6700688.f1.docx]

**S1**: A comprehensive search strategy for each database

| **Database** | **Choosing search terms** | | **Searching with keywords** | | **Searching for exact phrases** | | **Using Boolean logic** | |
| --- | --- | --- | --- | --- | --- | --- | --- | --- |
|  | **Articles found in the initial search** | **Articles initially included** | **Articles found in the initial search** | **Articles initially included** | **Articles found in the initial search** | **Articles initially included** | **Articles found in the initial search** | **Articles initially included** |
| *Google Scholar* | 3,673 | 87 | 3,670 | 74 | 12,500 | 186 | 16,500 | 57 |
| *PubMed* | 9 | 5 | 9 | 2 | 7 | 3 | 6 | 2 |
| *Wiley* | 7,533 | 48 | 2,030 | 45 | 8,004 | 120 | 1,202 | 35 |
| *Scopus* | 1,786 | 12 | 639 | 13 | 5,929 | 65 | 83 | 7 |
| *ScienceDirect* | 2,844 | 17 | 992 | 9 | 8,457 | 84 | 403 | 24 |
| **Total** |  | 169 |  | 143 |  | 458 |  | 125 |
| **Overall** | 895 | | | | | | | |

- Excluded articles that did not follow PICOs schema and were not well-structured.
